# “Clozapine makes me quite drowsy, so when I wake up in the morning those first cups of coffee are really handy”: an exploratory qualitative study of excessive caffeine consumption among individuals with schizophrenia

**DOI:** 10.1186/1471-244X-14-116

**Published:** 2014-04-16

**Authors:** Lisa Thompson, Amy Pennay, Adam Zimmermann, Merrilee Cox, Dan I Lubman

**Affiliations:** 1 Neami National, 247 – 249 Rosanna Road, 3084 Rosanna, VIC, Australia; 2 Centre for Alcohol Policy Research, Turning Point, Eastern Health, 54-62 Gertrude St, 3065 Fitzroy, VIC, Australia; 3 Centre for Health and Society, School of Population and Global, University of Melbourne, 3052 Parkville, VIC, Australia; 4 Turning Point, Eastern Health, 54-62 Gertrude St, 3065 Fitzroy, VIC, Australia; 5 Eastern Health Clinical School, Monash University, 3168 Clayton, VIC, Australia

**Keywords:** Schizophrenia, Psychosis, Caffeine, Qualitative, Australia

## Abstract

**Background:**

Research has shown that individuals with schizophrenia use caffeine at higher rates than the general population; however, no qualitative research has been undertaken investigating problematic caffeine use and its effects on this population. This article explores the role of caffeine consumption in the lives of people with schizophrenia through a narrative analysis of the attitudes and beliefs associated with this practice, and how these, in turn, influence caffeine consumption.

**Methods:**

A qualitative study was undertaken with individuals who had previously scored in either a ‘moderate’ or ‘high’ risk category for caffeine use on the Alcohol, Smoking and Substance Involvement Screening Tool (ASSIST). In-depth interviews were undertaken with 20 individuals, and transcripts were analysed thematically to identify prominent perspectives.

**Results:**

Consistent with previous literature, participants’ caffeine consumption was driven largely by its stimulating properties; however, participants also identified ‘cravings’ as an important motivating factor. Participants’ behaviours related to caffeine consumption seemed to be tempered by their previous experiences of consumption; if participants had experienced positive effects such as alertness or relaxation in the past, their use was maintained at a similar level or increased. Conversely, participants who anticipated negative consequences often altered their patterns of caffeine consumption; for example, by substituting caffeinated drinks that minimised or ceased their experience of negative side effects for those that directly caused such impacts. Overall, participants largely identified caffeine consumption as a highly meaningful activity, which provided structure to their day and facilitated opportunities for social interaction.

**Conclusions:**

The inconsistencies between individuals’ beliefs about their health and the actual risk of harm associated with health-related behaviours present significant and ongoing challenges for the implementation of relevant and effective strategies for health promotion among individuals diagnosed with mental illness. As a starting point, it would be worthwhile for services engaging with people diagnosed with mental illness, and in particular schizophrenia, to consider implementing caffeine-related health literacy strategies to educate consumers about the risk of excessive caffeine consumption and the interactions between caffeine and antipsychotic medications.

## Background

Caffeine is the world’s most widely used stimulant and is perceived to have a number of positive effects, including reduced fatigue, relaxation, and increased alertness [1]. Caffeine use of 500 mg per day and over (four to seven standard cups of coffee, or seven to nine cups of tea) is considered a ‘significant health risk’ and has been associated with the exacerbation of symptoms of mental illness [2,3]. Further, a significant relationship between caffeine consumption and dose of antipsychotic medication has been identified [4,5], with some studies suggesting a link between chronic caffeine consumption and the likelihood of experiencing negative side effects from medication [6].

In line with these findings, the suggestion that individuals with a mental illness consume caffeine at significantly higher levels than the general population is particularly concerning [6-8]. Larson and Carey [9] reported that their sample of psychiatric outpatients with a diagnosis of schizophrenia consumed seven times more caffeine than the general population, whereas other studies have reported mean daily caffeine intake among people with schizophrenia of between 400 - 471 mg [6,10]. In comparison, Brown and colleagues [11] reported a main daily caffeine intake of between 288 – 426 mg in a random sample of 481 adults from the general population in Ontario, Canada.

A recent study by Zimmermann and colleagues [12] explored substance use among consumers of an Australian community mental health service and found high rates of potentially problematic caffeine consumption. Zimmermann et al. [12] reported that 78.9% of participants were placed in a ‘moderate’ risk category and 7.4% in a ‘high’ risk category based on their caffeine use according to the Alcohol, Smoking and Substance Involvement Screening Tool (ASSIST) [13]. Overall rates of caffeine use were higher than all other substances screened as part of the study, including tobacco, alcohol and cannabis [12]. Within this study, problematic caffeine use was also found to be more common in individuals with a diagnosis of schizophrenia, as compared to other diagnoses. However, a limitation of the study was a lack of detailed information gathered on the specific behaviours of individuals related to their use of caffeine and in-depth exploration of the motivations and context framing their caffeine consumption.

Various theories have been proposed to explain elevated levels of caffeine consumption among individuals with a serious mental illness; including self-medication to treat symptoms such as anhedonia, drowsiness, and issues with concentration [14]; and to counteract the side effects of antipsychotic medications which can present in a similar way to the abovementioned symptoms [9]. However, few studies have explored caffeine use among individuals diagnosed with mental illness living in the community. We identified only one previous qualitative study of the use of legal drugs by individuals diagnosed with a range of mental illnesses, in which focus groups were conducted to explore the function of substances that included caffeine, nicotine and alcohol [2]. While the authors reported that psychiatric outpatients in their sample consumed caffeine to enhance arousal and to counteract the side effects of medication, such as drowsiness and dry mouth, the authors did not specifically focus on the experiences of individuals with schizophrenia.

The current study was undertaken to better understand the nature of caffeine use and its effects among individuals with schizophrenia using a qualitative approach. Qualitative designs are highly useful in exploratory studies with minimal previous research to draw upon [15]. The data present a first-person phenomenological account of caffeine consumption to better understand the subjective meanings associated with caffeine use among this group [16]. We explore qualitative differences in how participants experience and derive meaning from caffeine consumption, including individuals’ perceptions of their capacity to make changes to these behaviours, and describe the divergence of attitudes about, and motivations for, caffeine consumption through participants’ construction of a larger dialogue around the actual and potential harms of regular caffeine consumption.

## Methods

The current study was conducted across 10 Neami National sites operating in Melbourne, Victoria. Neami National, a community managed mental health organisation, provides rehabilitation and recovery support for people with a serious mental illness. A mixed methods design was implemented which involved the administration of a caffeine use questionnaire and in-depth interviews with a subset of the larger sample. This article draws on the findings of the in-depth interviews.

Consumer information was extracted from Carelink + (the internal consumer database) to identify consumers who had previously received a clinical diagnosis of schizophrenia or schizo-affective disorder (based on information from the referring clinician), and whose caffeine use scored in the categories of ‘moderate’ or ‘high’ risk according to ASSIST criteria [13]. The ASSIST is used routinely in clinical settings to indicate the presence of abuse and dependence symptoms and substance-related problems, supporting treatment outcomes by identifying individuals who might benefit from further assessment and intervention [17]. The ASSIST is routinely administered to all consumers across Neami National as it identifies a range of problems associated with substance use including acute intoxication, regular use, dependence or ‘high risk’ use and injecting behaviour. A Specific Substance Involvement (SSI) score is calculated which indicates the level of risk associated with the use of a particular substance, with high risk scores indicating that an individual is at high risk of dependence or is currently dependent on the substance, and is likely experiencing a range of issues as a result of their substance use [13].

Those consumers who were deemed eligible to participate based on their SSI scores were invited to complete the caffeine use questionnaire and undertake an in-depth interview with the research team after providing informed consent to participate in the study. The questionnaire administered by Neami support workers was designed to collect demographic information including information pertaining to comorbid disorders. This information was self-reported by participants; no clinical assessments were completed with participants to corroborate this data. The questionnaire also collected detailed information about individuals’ caffeine consumption patterns, their beliefs about the short and long-term risks associated with caffeine and their general health. In total, caffeine use questionnaires were completed by 59 individuals with schizophrenia or schizo-affective disorder between March and June 2012, and 40 individuals consented to be contacted for an in-depth interview, of which 20 were undertaken. Those who chose not to participate in interviews did so for a range of reasons, including experiencing acute illness or crisis; perceiving the questions as being too invasive; and reporting that they were not currently consuming as much caffeine as they had been previously.

Interviews were undertaken by LT and AZ, who both had no pre-existing relationship with participants. Interviews were semi-structured, and interviewers began by asking participants to describe a ‘typical day’ involving caffeine use, including frequency, amount and types of caffeinated drinks, when they used caffeine (i.e. shortly after waking; only during the day; all day; only at night), and whether they combined caffeine with other substances e.g. food, alcohol, cigarettes. If participants did not spontaneously provide the following information while describing a typical day of caffeine consumption, they were prompted to discuss: their reasons for consuming caffeine; experience of positive or negative side-effects both short- and long-term; attempts (if any) to decrease or cease consumption; perceptions of risk related to caffeine use, and perceived risk of caffeine use compared to other stimulants. Interviews were conducted in locations of convenience to the participant, including private homes, a local café, or their Neami site. By the twentieth interview, a saturation point had been reached where very little new information was being elicited, and so a decision was made to discontinue interviewing [18]. The interviews were recorded on a digital recorder and transcribed verbatim by an external organisation.

Thematic analysis was deemed the most appropriate method for interpreting interview data as themes can be constructed inductively and without reliance on a pre- determined theoretical paradigm, thus making it highly useful in exploratory studies with minimal previous research to draw upon [15]. Analyses were conducted using NVivo 9 (QSR International, Victoria, Australia) by three researchers (LT, AP, AZ). At least two researchers coded each interview, and thematic categories were firstly constructed inductively from interview data by all three researchers independently of each other [19]. Following the analysis process, LT AZ and AP met to compare their coding of the themes. The minor differences identified were discussed and negotiated until consensus was reached.

During analysis, it became apparent that caffeine consumption behaviours were underpinned by participants’ beliefs of the risk of harm associated with caffeine use. The following sections explore in greater detail the nature of caffeine consumption among participants and the ways in which their beliefs and expectations about caffeine use shaped their behaviour.

### Ethical considerations

The project had ethical approval from the Eastern Health Human Research Ethics Committee. Participants were informed about the purpose of the study, their right to refuse to participate without any adverse impact on their support or relationship with the organisation, as well as measures to ensure confidentiality; including using a numerical ID and removing any personal or identifying details from their interview transcripts. After this explanation, participants provided written consent to participate in the study.

## Results

### Description of the sample

Participants were aged between 29 and 61 years, with an average age of 42 years; seventeen men and three women took part; and 17 participants were born in Australia. The three participants born outside of Australia reported their countries of origin as Vietnam, England and Italy. None of the sample participants identified as being Aboriginal or Torres Strait Islander. Two participants had a diagnosis of schizo-affective disorder, and eighteen had a diagnosis of schizophrenia. The main antipsychotic medications that participants reported being prescribed were clozapine, olanzapine and paliperidone.

Forty percent of interview participants reported experiencing current issues with alcohol and/or other drugs, with 12 identifying as regular smokers, smoking an average of 21 cigarettes per day (eight of these individuals reported smoking a pack of 25 or more per day). In relation to other substance use as identified by SSI scores on the ASSIST, 15 participants were identified as being in a ‘moderate’ or ‘high’ risk category for tobacco (11 and four respectively); six in a ‘moderate’ or ‘high’ risk category for alcohol (four and two respectively); two in a ‘moderate’ risk category for cannabis; and one in a ‘moderate’ risk category for opioids. This is consistent with previous studies that suggest that a significant proportion of people diagnosed with serious mental illness have comorbid substance use disorders [20].

Participants’ SSI scores for caffeine revealed that the majority fell into a ‘moderate’ risk category (eighteen people), while two participants were in a ‘high’ risk category. These scores were highly representative of the larger sample that completed caffeine use questionnaires. Other than issues relating to substance use, participants did not report any co-morbid mental health disorders.

### Patterns of caffeine consumption

For the majority of participants, caffeine consumption began in the morning shortly after waking and continued throughout the day. More often than not, participants began their mornings by consuming either coffee or tea. Caffeine consumption in the morning was particularly meaningful for participants and was framed as a “rich ritual” that enabled individuals to ease into their day. Only one participant drank caffeine almost exclusively in the evenings. On average, participants consumed 2.4 different types of caffeinated drinks in any given week, including: Coca-Cola (fifteen people), instant coffee (twelve), tea (six), brewed coffee (four), iced coffee (three), and energy drinks (two). Eight participants reported consuming three or more different types of caffeinated drinks in any given week.

### The place of caffeine in participants’ lives

Two distinct groups of caffeine consumers emerged from the interviews. The first group saw their caffeine consumption as a trivial issue and did not see the reason for the strong focus of questioning about caffeine. For example: “I don’t know why [I drink caffeine], I think just boredom and things like that”. The second group were much more aware of their caffeine use and were able to articulate their reasons for use. These participants described the important role of caffeine in their lives and had clearly reflected on their caffeine use in the past. For example, one participant referred to caffeine as “food for the soul”, whereas another considered it “one of the simple pleasures of life”. Many of the second group’s narratives described the ritualistic nature of their caffeine consumption. More often than not, it was framed as a habit that had developed over a considerable period of time, often beginning in childhood. Some participants could not imagine life without caffeine and discussed their caffeine use as essential for daily functioning. These participants framed their caffeine use more in terms of addiction or dependence.

### Dependence and craving

While no direct measures of abuse/dependence were administered among interview participants, the average caffeine consumption of coffee drinkers within the sample was 523 mg per day (which is considered a ‘significant health risk’ [2,3]), and half of participants reported feeling that they had minimal control over their caffeine consumption, with some indicating an inability to live without caffeine. For example:

“I was vampirical [sic] on coffee like just drinking it like it was my life source”.

“I don’t miss cigarettes either, but coffee, I have to have coffee…I don’t think I could live without coffee”.

“I’d be lost without my Coca Cola. I couldn’t survive without it…I can’t get off to sleep without it…I need to have a certain amount each day to get me through”.

Other participants were less emphatic in their responses, but still expressed feelings of needing or wanting caffeine:

“I just felt like I needed it…If I didn’t have a cup of coffee in the morning or something I felt a bit dizzy”.

“It’s addictive and I just enjoy it…I bought tea a few weeks ago and I tried to replace a tea for a coffee, I found it very hard…I’d like to not be as dependent on it, but it’s just a natural thing just to wake up, put the jug on, make a cup of coffee”

For the abovementioned participant, the need to consume caffeine overrode the sub- optimal conditions of her accommodation in which to prepare caffeinated drinks:

Participant: I’ve tried cutting down, but that’s a waste [of money]… and the thought to have it was just too strong. So I wake up at say nine, have breakfast then I go straight to my room and I have two or three coffees. Interviewer: Is there a reason why you drink two or three in a row as opposed to just one?

Participant: I think because I get water from the hot water tap, it’s not really hot water, it’s lukewarm.

Interviewer: OK is there any reason why you don’t use a kettle? Participant: We’re not allowed to have them…for safety reasons.

### Benefits of caffeine use

The stimulating properties of caffeine were one of the primary motivations for morning consumption; many people enjoyed the “boost” and the “buzz” from caffeine. One participant mentioned that “like everyone I’m a bit lethargic waking up and all that sort of stuff, but then I put on the kettle as soon as I wake up”. This appeared to be an experience shared by the majority. Some participants used caffeine for relaxation. For example, one participant almost exclusively consumed coffee in the evenings as a way to “chill out” in the same way that another person might drink alcohol: “I wouldn’t call myself a coffee, caffeine addict, but yeah I don’t mind having a couple of…oh like probably in the night, I don’t know five cups or something”. Others consumed caffeine throughout the day and used caffeine as a reward or used their caffeine break as a way to disrupt their routine, for example:

“It gives me a bit of time out…A break from the pressures of the day”.

“When I’m working I have a coffee after about two or three hours, just to fill in, as a break”.

“Yeah it’s sort of like a luxury thing, yeah you just feel like it”.

Some participants were staunch advocates for caffeine and looked for ways to justify their use. For example, one participant said they had “heard on the news the other day that people who drink coffee live longer…I thought I’m coming to see you today, I’ll remember to mention the fact that coffee drinkers live longer”.

The majority of respondents reported consuming caffeine, at least in part, for its pleasurable taste. In particular, the sugary taste was identified by participants as an important factor in their enjoyment of caffeinated drinks. Some participants chose soft drinks, instant coffee and iced coffee over brewed coffee for this reason:

“You can’t kill it [brewed coffee] with sugar like the other stuff…I prefer instant, the instant stuff’s smaller, you know more smooth and creamy as opposed to the others”.

“[Iced coffee is] a bit sugarier and tastier [than instant coffee] and moorish is probably the right word for it”.

“I like the sweetness in the Coca Cola, the sugar and that”.

However, some participants also continued to consume caffeine even though they reported not enjoying the taste; for two individuals the perceived benefits of caffeine overrode their distaste for the drink. There was a strong link between drinking caffeine and smoking cigarettes, consistent with previous research [7,21,22]; irrespective of whether participants were current or former smokers, there was a general sense that caffeine and nicotine “go hand in hand…combined, they both taste good”. Previous literature suggests that caffeine ingestion increases reinforcement from nicotine [21-23], and that caffeine use may be a contributing factor in the onset, maintenance of or relapse to tobacco dependence [21], which may provide some explanation for the high rates of concurrent caffeine use and smoking among participants.

The final motivation for caffeine use cited by participants was the social aspect of consumption. It is well documented within the literature that people with a diagnosis of psychotic illness experience issues around interpersonal relationships and loneliness [24-26] so it is unsurprising that almost half of participants cited the social aspects of caffeine consumption as being a primary motivator for their behaviour:

“[Drinking caffeine during childhood] was about bonding with my mum…having a coffee and a biscuit and stuff”.

“Once or twice a week I go for a coffee with my friend… I usually ring up a friend and go for a coffee and have a chat”.

“It’s just such a nice social thing. The Italians do it and the Greeks do it…it’s like when you go to someone’s house, do you want a cuppa? My friend comes each night and we have a coffee, a biscuit, then dinner…People meet and greet over coffee, friends, old friends and family, you know”.

“In the hospital…with people with mental illness it’s a sort of a starting point, it’s a social thing, you know cigarette, bottle of coke and when you speak to other people with mental illness you know it revolves around cigarettes and Coca Cola”.

A number of participants couched their caffeine consumption in terms of caffeine’s perceived social acceptability compared to other substances such as alcohol or cannabis. It appeared as though social perceptions about caffeine played a significant role in participants’ decisions about consumption, particularly around being seen to be “trendy” or “fashionable” by others. The desire to be perceived in a certain way through the consumption of caffeine within a social context might relate to the challenge of social inclusion for people experiencing enduring mental illness.

### Perceptions of caffeine compared to other drugs

Participant’s perceptions of their caffeine use were influenced by the social positioning of caffeine use as a substance they perceived as less harmful than other licit and illicit substances, namely alcohol, cannabis, cocaine, methamphetamine and heroin, but most still considered caffeine to be a stimulant, “like a drug”:

“Amphetamines are far stronger, but it [coffee] does do that, it makes you race a bit…The coffee sort of makes you feel like you’re on speed”.

“I do perceive it as a stimulant but I wouldn’t perceive it as something that could create that much harm unless it was taken in extreme amounts”.

“I don’t see it as a big deal… I know a lot of people who have no addiction or anything to it”.

“I don’t even think it comes even close [to illegal drugs]… it’s legal, it’s normal as far as I’m concerned. Yeah it might have you know the side effect of keeping you awake or more alert whatever they’re saying about coffee, but I would never even put it in the same category as drugs like that”.

“Well it’s definitely not the worst [substance]…things like alcohol and other drugs are a lot more damaging than caffeine”.

These perceptions of caffeine compared to other substances seemed to temper participants’ responses about their expectations of the risk of harm associated with consuming caffeine. With the exception of a few individuals who had experienced significant impacts on their physical or mental health, participants generally regarded caffeine as one of the ‘safer’, more socially acceptable substances that they could be consuming.

### Negative effects of caffeine

Few participants expressed concerns about their caffeine consumption in terms of either short or long-term harms. However, when prompted, a number of participants reported experiencing negative side effects from what they considered excessive caffeine consumption, including experiencing dry mouth; insomnia; bloated stomach; blurred vision; anxiety; hallucinations; nausea, and vomiting. For most, the experience of these side effects was not significant enough to elicit a change in their consumption. In fact, many participants had not experienced negative side effects and did not believe that caffeine was harmful. Those who stated concerns about caffeine were most often in relation to the consumption of soft drink as opposed to coffee.

A small group (three people) perceived caffeine to be a potentially harmful drug and framed their caffeine consumption in terms of attempts to cut down or quit. Some participants explicitly discussed their recent efforts to cut down or cease their caffeine consumption; however, almost all of these individuals had returned to their previous levels of consumption. For example:

Participant: If I do things like go to the gym after I’ve had some tea like I did yesterday, it affected me after the gym. I start hearing voices and get a fair bit of anxiety and stuff like that… I don’t know whether I’m addicted to it or…I also like the taste of Pepsi Max so much I, I’d like to still be able to drink it, but I’ve cut down since [realising it was contributing to psychosis] I haven’t had any Cola for a little while… Interviewer: So when you say you don’t know if you’re addicted to it, what would… How do you think that that would look?

Participant: I guess you’d, you’d want to be drinking it every day…Which I’m doing pretty much, but I mean because of the psychosis yesterday I didn’t have any.

This participant had also ceased consumption of coffee as it was contributing to his experience of psychosis:

Interviewer: And what about coffee, you’ve never liked the taste of coffee?

Participant: No I love the taste, but I just went off it because it was affecting me [increasing frequency of psychosis] too much…It sort of got stamped in my brain I guess that if it was coffee then no matter how strong it was, whether it was normal coffee or a decaf, it just stamped in my brain that it was going to affect me.

One participant recounted that their high levels of caffeine consumption had affected their physical health to the point that they had recently been admitted to hospital, but had since been able to decrease their consumption. This participant did not specify what their physical health condition was, but stated that:

“I was drinking it [instant coffee] all day…right up to about eight o’clock at night and I couldn’t control myself. I just for some reason couldn’t control myself and that so eight o’clock at night would be my last coffee and then during the night I felt like I couldn’t sleep because of it…I was also drinking probably four or five 1.25 litre [bottles] a day…I ended up in hospital for five days, because the fizzy drinks blew, blew my tummy up a lot…I had a tube up my nose and back down my throat and yeah”.

The experiences outlined above represented the most significant impacts on participants and were indicative of the severity of risk of harm required to warrant a decrease or cessation of caffeine consumption.

Most participants stated a preference for caffeinated drinks with high sugar content or that tasted ‘sugary’; however concurrently expressed an awareness of the potentially harmful effects of sugar on their physical health. A handful of participants had changed their consumption practices as a result of this perceived risk:

“Sometimes I had one 1.25 Litre bottle of normal Coke and I thought oh this tastes great and hang on a sec I shouldn’t have drunk that because of the sugar and yeah I changed. Yeah that full on sugar rush is not very good for me”.

In general, though, participants were uninterested in changing their level of sugar consumption unless a trusted authority e.g. General Practitioner made a recommendation to that effect.

### Caffeine and interactions with medication

Some participants consciously reflected on the interaction between caffeine use and their medication. For example, one participant suggested that drinking caffeine “will actually help break down medication…research says that four or five coffees a day actually help your liver function”. This participant reported that:

“When I was taking hard tabs [of antipsychotic medication], I’d have a couple of coffees to whack it down [sic] in my bloodstream…If I was taking a lot of hard tablets I would probably consume more Coca-Cola or coffee to break it down”.

This participant believed that caffeine consumption improved their liver function, and believed that the stronger the dose of medication they were taking, the more caffeine they would need to consume to metabolise it. Further, other participants emphasised the importance of caffeine consumption to counteract the sedation that they experienced after taking medication: “Clozapine makes me quite drowsy, so when I wake up in the morning…those first cups of coffee are really handy”. Others had noticed that caffeine consumption had an impact on the effectiveness of their medication, but found it difficult to verbalise the specific interaction with caffeine:

“It’s probably not the best habit to have, drinking too much [coffee] because I do think it does keep you awake far longer than you should…I don’t know for certain how it effects the medication, but I do think that there’s…it’s a definite yes as far as the effectiveness of it [medication]… Because like I said, when I have taken it, even when I’m exhausted I’ve already probably drank four or more coffees than I probably usually would and there’s still that agitation and not as easy to get to sleep. Whereas if I stick to what I usually do, within half an hour of taking the medication it’s pretty much guaranteed that I’m gonna [sic] be falling asleep”

“I’ve been on Seroquel and stuff like that and when I was drinking coffee and taking that medication, it triggered like…it had a very stimulating effect, I thought it was maybe to do with combining the medication and coffee”.

“You see with clozapine it puts me to sleep regardless of the caffeine I drink during the day…but I was also told, the doctor told me once that drinking Coca Cola can affect the levels of clozapine in your blood”.

Even though participants’ reflections did not all concord with previous research around interactions between caffeine and antipsychotic medication, it was noted that participants were aware of psychological and physiological changes associated with medication use and caffeine consumption, even though close to eighty per cent of the larger sample of 59 participants stated that no-one had told them about possible interactions. The suggestion by some participants that they noticed changes in the effects of their medication based on their caffeine consumption indicates that further research into these interactions is warranted, particularly in light of recent clinical studies, which provide evidence of a robust effect of caffeine on decreasing serum clozapine levels among some individuals [27,28].

## Discussion

To our knowledge, this article is only the second qualitative study aimed at understanding the role that caffeine plays in the life of people with serious mental illness, and the first qualitative study attempting to understand caffeine use among people with schizophrenia. Our analysis revealed that the patterns of, and motivations for, caffeine consumption among our sample were varied. In particular, two divergent narratives emerged from the interviews, with participants offering opposing viewpoints about caffeine’s potential for harm. The first group constructed their caffeine consumption as a trivial issue, one that posed no risk of harm to their physical or mental health. Participants in this group either perceived their caffeine use as a non-issue or cited perceived benefits and/or positive effects of consuming caffeine. Accordingly, this group reported little to no desire to make changes to their caffeine consumption. On the other hand, a smaller group acknowledged either potential or actual harms that they had experienced as a result of caffeine consumption, most often in relation to soft drinks (as opposed to coffee). This group also acknowledged the benefits of caffeine use, but were more likely to frame their consumption in terms of addiction or dependence.

Participants’ behaviour appeared to be driven by expectations about caffeine consumption based on previous experiences and perceived feelings of control over their patterns of use. If participants had experienced positive effects from caffeine consumption in the past, such as relief from boredom, or increased alertness, participants reported positive beliefs about caffeine consumption and their use was maintained and often increased, as they did not perceive themselves to be at serious health risk. This seemed particularly relevant for participants who framed their use in terms of dependence: consuming caffeine at consistent or increasing levels gratified feelings of ‘needing’ or ‘wanting’ caffeine, or mitigated side effects ostensibly as a result of caffeine withdrawal, thus perpetuating the behaviour through the anticipation that consuming caffeine would prevent such negative consequences in future.

Conversely, participants who anticipated negative consequences often altered their patterns of caffeine consumption. The degree of behaviour modification appeared to vary according to the severity of the harm that participants anticipated occurring as a result. Participants who had previously ceased consuming a type of caffeinated drink due to anticipated harm often substituted other types of caffeinated drinks. For example, one participant was hospitalised as a result of excessive soft drink consumption and began drinking coffee after discharge from hospital as this form of caffeine did not cause gastrointestinal upset. As outlined above, one participant stopped drinking coffee and cola as these drinks exacerbated their psychosis and began drinking tea as it had less of an impact on their symptoms. In this respect, participants who anticipated negative consequences from caffeine altered their behaviour in line with their previous experience to achieve an ‘ideal’ level of caffeine consumption that they anticipated would maximise positive affect and minimise harm. This also appeared to be true of participants’ perceptions of risk associated with sugar consumption, although participants seemed more willing to abide by the advice of a trusted professional if they believed their health to be at risk.

Overall, participants’ decision making around caffeine consumption (based on the risk of harm and perceived social desirability) appears similar to the hypotheses for these behaviours offered by previous studies [2,9]. These studies suggest that individuals with a mental illness largely consume caffeine to self-medicate and reduce side effects of medication. Participants from both studies identified ‘increased alertness’ and ‘taste’ as a factor that influenced their caffeine consumption. Significantly more participants from the current study than the study by Carey et al. [2] identified cravings (expressed as needing or wanting caffeine) as a motivating factor for consumption. It is unknown whether these motivations are consistent with those of the general population, as we were unable to identify any qualitative studies that have explored these factors among community samples.

Further, that participants noticed and described an interaction between caffeine consumption and antipsychotic medications corroborates the findings of clinical studies which suggest that caffeine can impact on serum clozapine levels [27,28]. Further research would better illuminate the complex interactions between caffeine consumption and antipsychotic medications, especially given the prevalence of (often excessive) caffeine consumption among this group.

Participants expressed a preference for, and a tendency to consume caffeinated drinks with high sugar content; and most often combined caffeine with cigarettes. Participants’ comments about the sugar content of their preferred caffeinated drinks were consistent with previous literature indicating that people diagnosed with schizophrenia consume sugar at levels significantly higher than World Health Organisation recommendations [29,30].

These findings, when considered together, contribute to the body of evidence suggesting a link between sugar, tobacco and caffeine [4,21-23], and as such, future research could investigate the influence of sugar and tobacco smoking on caffeine craving, particularly among individuals diagnosed with mental illness.

### Limitations

Intentional sampling was used to recruit participants; if participants had been selected randomly, this may have elicited greater variation in results and thus represented a more diverse range of perspectives. Further, since participants were recruited from one specific service type within a single state, it is likely that the findings cannot be generalised to the wider population. Reliance on participants’ recall of caffeine use, rather than objective measures, potentially limits the validity of the data. Consideration should be given to the potential for participants’ short-term memory to be compromised by impairments which are commonly held features of schizophrenia [31]. To overcome this issue, future research might benefit from utilising ecological momentary assessment tools to enable participants to report on symptoms, affect and behaviour in close to real time [32].

Nevertheless, the current study was largely explorative in the sense that only one previous study could be identified that targeted a similar population; even then, the level of detailed analysis was insufficient to draw parallels with the findings from the current study. Given that previous research into the short and long-term impact of caffeine consumption has elicited inconsistent findings, further research to assess the risk of harm associated with interactions between psychotropic medications and caffeine is warranted.

## Conclusions

Numerous studies have linked excessive caffeine consumption with negative effects on physical and mental health [2-6]. However, as responses from participants within the current study indicated, the inconsistencies between individuals’ beliefs about their health and the actual risk of harm associated with health-related behaviours present significant and ongoing challenges for the implementation of relevant and effective strategies for health promotion among individuals diagnosed with mental illness. As a starting point, it would be worthwhile for services engaging with people diagnosed with mental illness, and in particular schizophrenia, to consider implementing caffeine-related health literacy strategies to educate consumers about the risk of excessive sugar and caffeine consumption and the interactions between caffeine, tobacco, sugar and antipsychotic medications. Given the number of participants within the current study who strongly endorsed the benefits of caffeine consumption, the challenge of engaging consumers in strategies around modifying caffeine consumption will no doubt be ongoing. Nevertheless, addressing excessive caffeine consumption is an important component of improving long-term physical health outcomes for individuals diagnosed with serious mental illness.

## Competing interests

The authors declare that they have no competing interests.

## Authors’ contributions

All authors contributed to the design of the research project. LT and AZ carried out in-depth interviews. LT, AZ and AP undertook data analysis and AZ and AP validated the thematic categories generated by LT’s analysis. LT drafted the manuscript, and AP, AZ, MC, and DL provided editorial support and approved the final manuscript. All authors read and approve the final manuscript.

## Pre-publication history

The pre-publication history for this paper can be accessed here:

http://www.biomedcentral.com/1471-244X/14/116/prepub
